# High frequency of intermediary alleles in the *HTT* gene in Northern Sweden - The Swedish Huntingtin Alleles and Phenotype (SHAPE) study

**DOI:** 10.1038/s41598-020-66643-0

**Published:** 2020-06-17

**Authors:** Jimmy Sundblom, Valter Niemelä, Maria Ghazarian, Ann-Sofi Strand, Ingvar A. Bergdahl, Jan-Håkan Jansson, Stefan Söderberg, Eva-Lena Stattin

**Affiliations:** 1 Department of Neuroscience, Neurosurgery, Uppsala University, Uppsala, Sweden; 2 Department of Neuroscience, Neurology, Uppsala University, Uppsala, Sweden; 3 Science for Life Laboratory (SciLifeLab), Department of Immunology, Genetics, and Pathology, Uppsala University, Uppsala, Sweden; 4 The Biobank Research Unit, Umeå University, Umeå, Sweden; 5 Department of Public Health and Clinical Medicine, Research Unit Skellefteå, Umeå University, Umeå, Sweden; 6 Department of Public Health and Clinical Medicine, Heart Centre, Umeå University, Umeå, Sweden; 7 Department of Immunology, Genetics and Pathology, Science for Life Laboratory, Uppsala University, Uppsala, Sweden

**Keywords:** Genetics, Neuroscience, Neurology

## Abstract

Trinucleotide (CAG) repeat expansions longer than 39 in the huntingtin (*HTT*) gene cause Huntington’s disease (HD). The frequency of intermediate alleles (IA) with a length of 27–35 in the general population is not fully known, but studied in specific materials connected to the incidence of HD. The Swedish Huntingtin Alleles and Phenotype (SHAPE) study aims to assess the frequency of trinucleotide repeat expansions in the *HTT* gene in north Sweden. 8260 individuals unselected for HD from the counties of Norr- and Västerbotten in the north of Sweden were included. DNA samples were obtained and analysis of the *HTT* gene was performed, yielding data on *HTT* gene expansion length in 7379 individuals. A high frequency of intermediate alleles, 6.8%, was seen. Also, individuals with repeat numbers lower than ever previously reported (<5) were found. These results suggest a high frequency of HD in the norther parts of Sweden. Subsequent analyses may elucidate the influence of IA:s on traits other than HD.

## Introduction

Huntington’s disease (HD) is caused by a CAG triplet repeat expansion, encoding polyglutamine (polyQ), in the huntingtin (*HTT)* gene on chromosome 4^1^. The disease is inherited in an autosomal dominant fashion and leads to motor, cognitive and psychiatric symptoms, with a variable age of onset influenced by the length of the CAG repeat. Expansions with more than 39 repeats invariably cause the disease during a normal lifespan, while alleles with between 36–39 repeats are considered to confer reduced penetrance and may cause disease in an individual depending on other genetic modifiers and/or lifestyle factors^2–5^.

The worldwide prevalence of HD is 5.5/100 000^6^, making it a relatively common monogenic neurological disorder. The highest prevalence is found in populations with European ancestry, while for instance African and Asian populations have a much lower prevalence. As genetic tests have been widely implemented for persons at risk of HD, effort has been made to ascertain the predictive value of test results, depending on the number of repeats^2^.

Especially challenging in the setting of genetic testing for HD is the “gray zone” interval of 27–35 repeats, referred to as intermediate alleles (IA), that do not cause HD, but due to instability may expand to cause disease in the offspring by anticipation^7^. Several hypotheses have been suggested to explain this phenomenon, including impaired DNA mismatch repair systems^8^.

Rare cases have been reported where the classical phenotype has presented in individuals with repeat expansion numbers in the intermediate interval^9,10^. This may potentially be mediated by loss of interruption between the CAG repeat and an adjacent polymorphic proline tract (CCG)^11^.

Reduced penetrance alleles and their pathological significance have also been studied in persons at risk of HD but not in the general population^12–14^. One study has described subtle but significant clinical signs when subjecting older IA carriers to clinical investigations aimed at grading motor and cognitive symptoms of HD patients^12^. Certain individuals may exhibit signs and symptoms also earlier in life^15^. In a sample of 50 individuals, an increased self-reported risk of suicide attempts was found^13^. There is also some data suggesting an increased lifetime risk of depression in individuals with IA^16^.

As commercial genetic screening tests are gaining use outside of the healthcare provider setting, it may be a question of time before the *HTT* gene (along with other monogenic disorders) is included in such panels. This would pose new demands on the healthcare providers to manage patients with genetic test results acquired without any family history of HD^17^.

Some groups have recently found a high prevalence of CAG repeats in the intermediate and reduced penetrance range in the background population^18^. There is also indication that the prevalence of HD, and frequency of new mutations, is correlated to the mean repeat number and prevalence of IAs in the general population. The national prevalence of HD in Sweden has not been calculated since the seventies^19^, but large regional variances has been reported more recently^20^. Establishing the frequency of IA may give us better data concerning the true prevalence of HD in this region.

The aim of this study is to describe the frequency of different CAG expansion lengths in the *HTT* gene in a population not selected for familial history of HD. In addition, these data will be used to establish a cohort enabling subsequent analyses pertaining to phenotypic variation in the length of the expansion in the *HTT* gene, including health effects.

## Materials and methods

**Figure 1:**
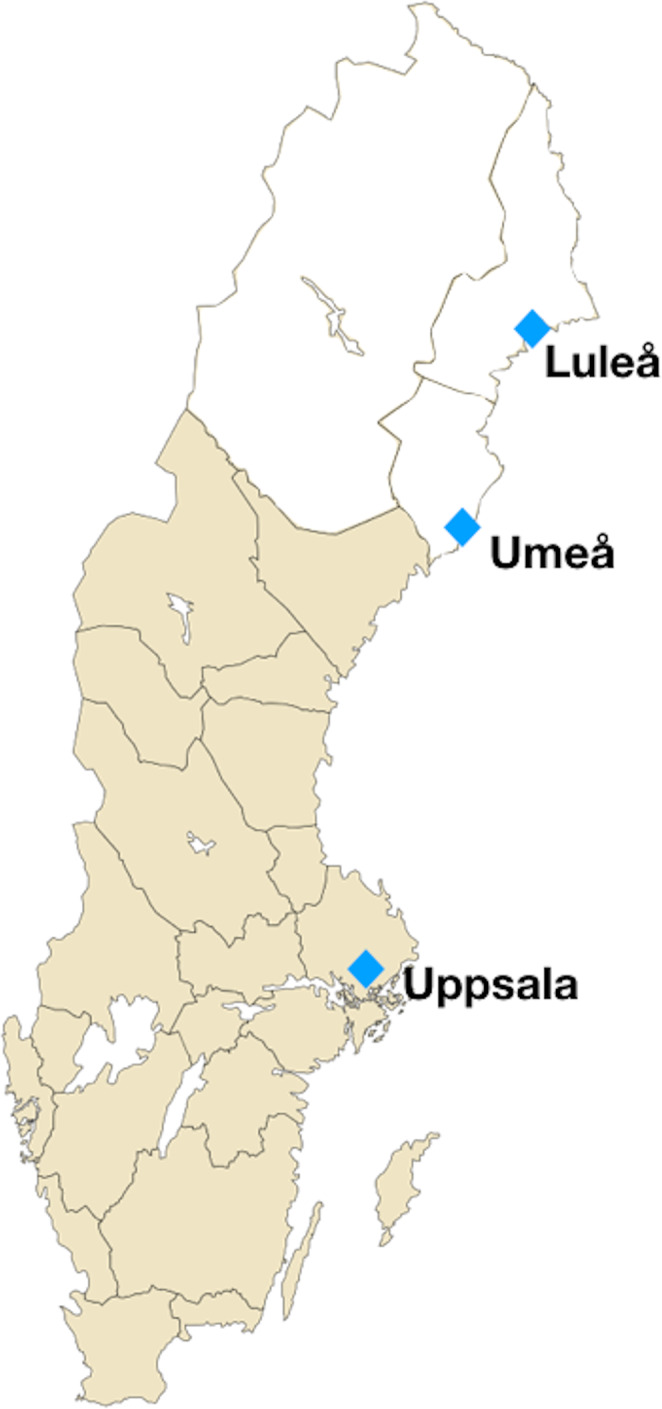
Map of Sweden. The regions from which persons were recruited to the NHSDS highlighted in white.

In the current study, we genotyped a sample of 8260 individuals from the population of the counties of Västerbotten and Norrbotten, Sweden, to measure the distribution of Huntingtin CAG repeat lengths. Västerbotten (pop 269 835) and Norrbotten (pop 250 497) are mainly rural areas with a few small cities (<50,000 inhabitants) and two larger urban centres, Umeå (pop 127 119) and Luleå (pop 77 000)(Fig. 1). The sample thus includes roughly 1.5% of the population of the region.

We used a prospective nested case control design with myocardial infarction (MI) cases and matched controls, established within the Northern Sweden Health and Disease Study (NSHDS)^21^. NSHDS includes individuals recruited from three large epidemiology projects; the Västerbotten Intervention Project (VIP)^22^, the Northern Sweden MONItoring of Trends and Determinants in Cardiovascular Diseases project (MONICA)^23^, and the Mammary Screening Program (MSP)^21^.

VIP was launched 1985 in Norsjö, a small municipality in Västerbotten, with the aim of reducing morbidity and mortality from coronary vascular disease (CVD) and diabetes^22^. VIP was successively implemented across the county (since 1991) and gradually integrated into ordinary primary health care routines (since 1995). Individuals at ages 30, 40, 50 and 60 years were invited to participate in risk factor screening and individual healthy life style counselling.

MONICA consists of randomly selected individuals aged 25 to 74 years from the counties of Västerbotten and Norrbotten who were invited to participate in a health study. The study started in 1986 and has been repeated 7 times with around 5-year intervals with new random samples of 2500 individuals each (the first 2 surveys invited 2000 individuals each)^24^. In addition, all cases with an acute myocardial infarction (MI) aged less than 65 years have since 1985 been validated and included in the MONICA register.

Mammography screening was offered to all women 50–69 years between 1995 and August 2000 and to all women 40–74 years between September 2000 and 2006 in the Västerbotten county and they were asked to report length, weight and smoking habits.

All individuals participating in MONICA, VIP and MSP donated blood samples for research purposes. These were stored in a biobank.

Questionnaire- and basic demographic data was collected at the time of blood sampling in the MONICA and VIP cohorts, which allows for a preliminary analysis of potential phenotypic traits mediated by IAs in the general population, as well as allowing us to identify possible selection bias before data is extracted from other registries.

The medical data collected at inclusion in the original cohort includes length, weight, body mass index (BMI), blood pressure, history of and current tobacco use, pharmacologic drug use, and basic laboratory measurements.

Demographic data includes age at inclusion, gender, marriage and relationship status, education level, country of birth, job status and more.

Questionnaires completed by the individuals in the cohort were SF-36, AUDIT, Cambridge physical activity index, Interview Schedule for Social Interaction (ISSI). A separate questionnaire designed for the MONICA-cohort addressed general physical activity. A list of all provided data is included as a supplementary file.

From the NSHDS, we included 2431 randomly selected cases with MI and two controls per case without known MI, matched for sex, age (±2 years), geographical area, cohort (MONICA, VIP or MSP), and date of health examination (±4 months). No link between HD and MI is currently known, and the cohort is thus considered unbiased concerning HD as well as possible confounding neurological disorders.

The data that was extracted for this cohort is cross-sectional, but many participants have donated repeat blood samples every decade (at age 40, 50 and 60) as well as undergone general health check-ups.

**Figure 2:**
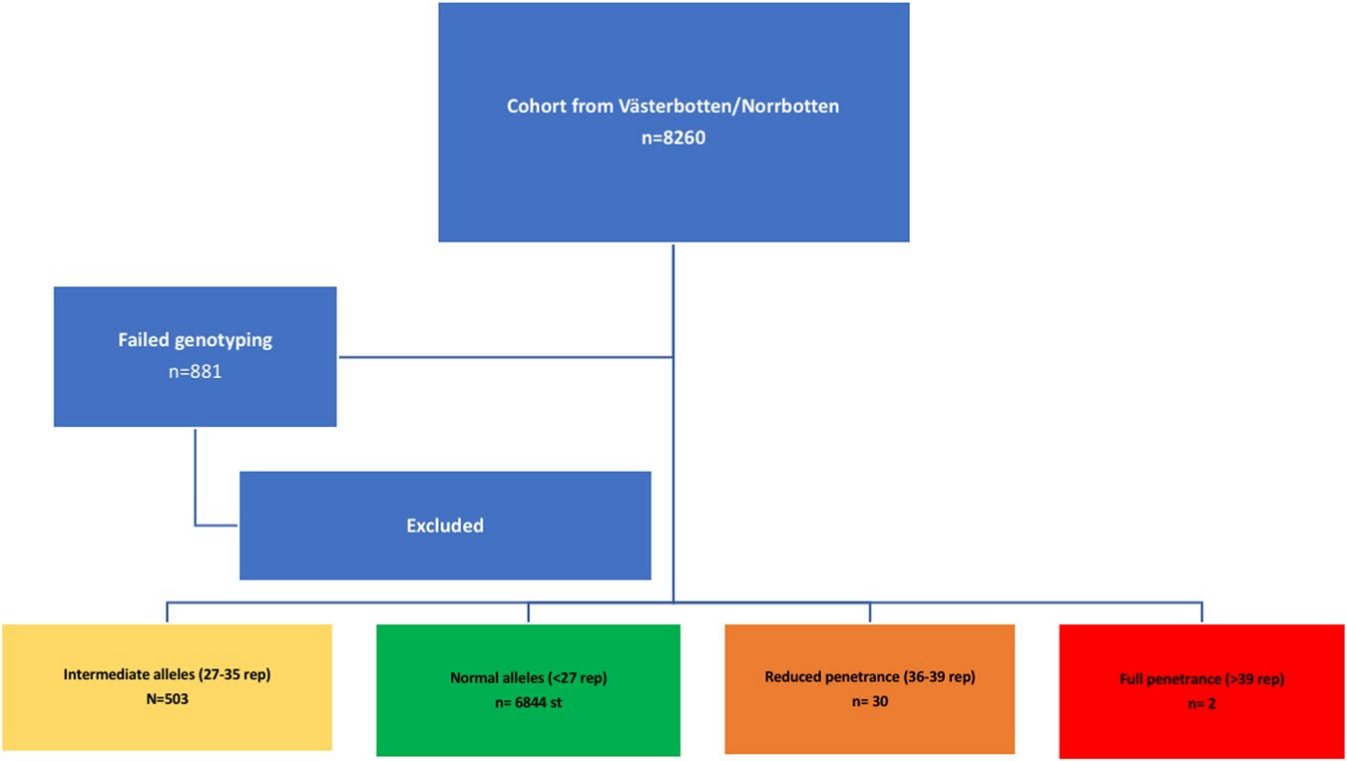
Overview of the inclusion process.

Figure 2 provides an overview of the inclusion process.

Analysis of the *HTT* gene CAG expansion was performed in all individuals, using polymerase chain reaction (PCR) by commercially available primers (Coriell Institute for Medical Research, NJ, USA)^25^. In total 8260 samples were collected, of which 881 samples (11%) were lost due to technical issues (fragmented or low concentration DNA, high guanine-cytosine content, small bubbles in the pump or blocks, etc). Complete data from both alleles of each sample were acquired from the remaining 7379 samples.

DNA samples were analysed for expansion size of the CAG repeat in the *HTT* gene at the NGI/SciLife Lab (Uppsala). The number of trinucleotide repeat was established by PCR analysis of the region including the CAG repeat, followed by fragment sizing through capillary electrophoresis at sufficient resolution to allow separation of alleles with one repeat difference. Control samples with well-defined repeat sizes (19/21, 17/36, 45/47, 22/65) were used for allele sizing and validation of the method.

In the original study population aimed at studying MI the cases were either included before or after MI (Fig. 2). Since inclusion after an MI (defined as a retrospective case) possibly could bias the variables of interest in our study, we checked whether the rate of retrospective cases differed between the IA group and the individuals in the normal allele size group, but no such effect was found. The rate of MI in the IA group was 30,28%, and the rate of MI in the control group was 33,33%, thus the hypothesis that the groups would be similar in that respect could not be rejected (Chi^2^ test, p = 0.160).

### Ethical considerations

Huntington’s disease is a devastating disorder for which there is currently no disease-modifying therapies.

Analysing the gene causing the disease unbeknownst to the individuals concerned is therefore fraught with ethical concerns. The research team received pseudonymized data with a serial number from the Biobank Research Unit at Umeå University. Anonymity is important, since persons included in the NSHDS reap no immediate benefits from knowing the fact that one carries a disease-causing or reduced penetrance allele in the *HTT* gene.

The one possible advantage of receiving this knowledge is the availability of prenatal testing and pre-implantation genetic analysis options when reproducing, but this benefit does not in our opinion outweigh the severe distress unsolicited information of HD carrier status would cause.

There is a slight risk that individuals could be identified by linking data from several different registries, which is addressed by the fact that the genotyping and data handling centre is located in a different region than the uptake area of the NSHDS.

Written consent was provided by all participants at study entry for the NSHDS, i.e. when the blood sample was taken, and this add-on study was approved by the Regional Ethics Review Board at Uppsala University and the Expert Committee of the NSHDS. The study was carried out in accordance with relevant guidelines and regulations.

## Results

The SHAPE study is the first study to investigate the phenotype associated with CAG repeat numbers of the *HTT* gene in the general population. Previous studies focused on participants related to HD patients, and therefore selection bias cannot be ruled out. Genetic modifiers contributing to development of clinical HD are likely to have been influential in these samples.

### High occurrence of intermediate- and reduced penetrance alleles in Västerbotten and Norrbotten

The frequency of intermediary alleles (IA) was 503 (6.8%) while 30 individuals (0.4%) had a reduced penetrance allele and 2 individuals had a full penetrance allele. Compared with previous studies^26,27^, this population has among the highest reported prevalence of IA:s, and numerically translates to the largest single cohort of IA:s to date. The frequency of reduced penetrance alleles was also strikingly high, 1/250 individuals. Three individuals carried two IA:s, while a further two carried one IA and one reduced penetrance allele. One of the individuals with a full penetrance allele also had one allele of intermediate length.

### General distribution of CAG repeat numbers

**Figure 3:**
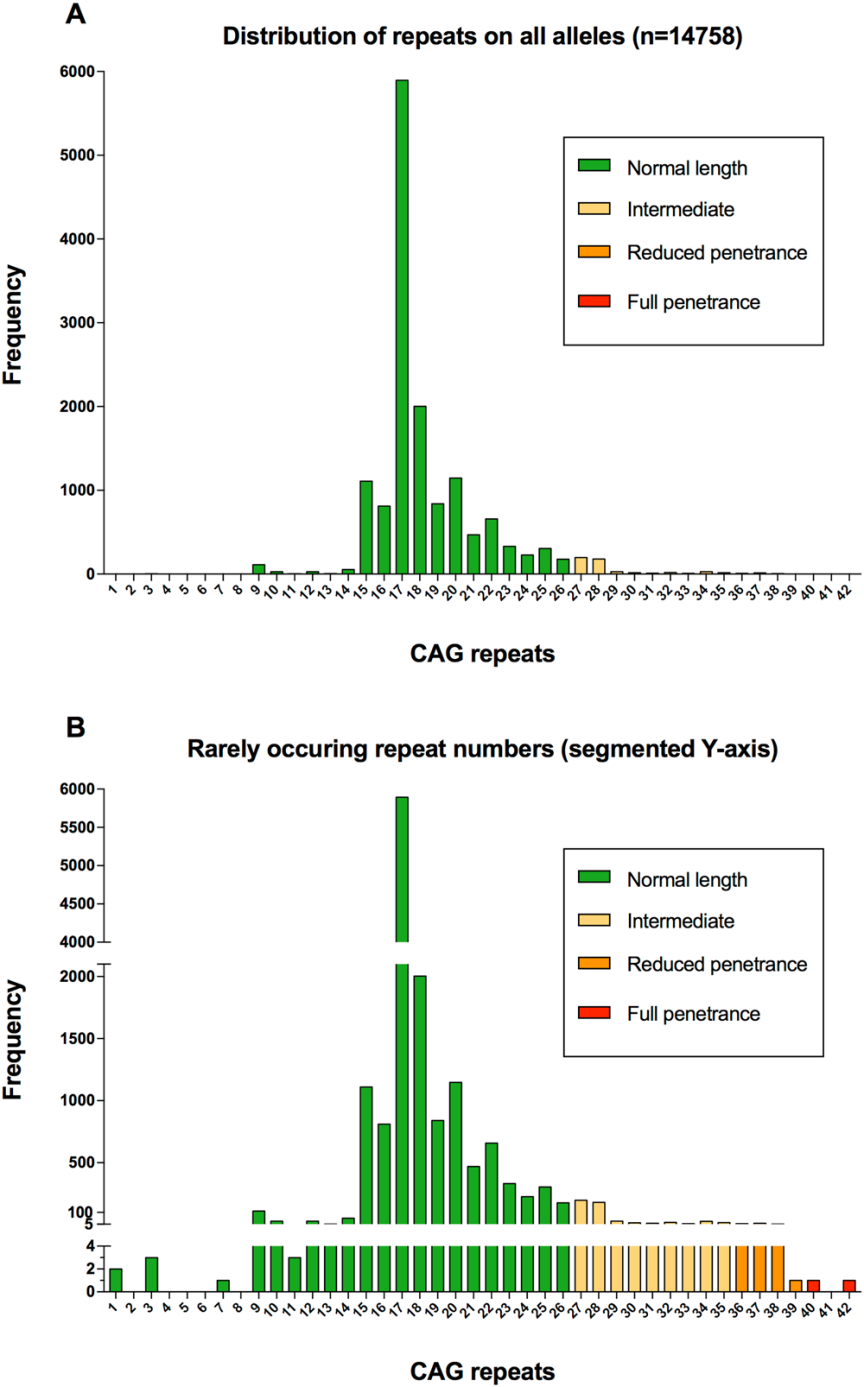
Distribution of HTT trinucleotide repeat length in the study. Note the distribution, with a pattern of normal distribution centered around a length of 17, which is the by far most common length. Also of note are a few individuals with repeat lengths in the disease causing spectrum (>39). Few individuals with hitherto unreported very short repeat lengths were also found.

The general distribution of repeats mimicked a normal distribution if the median number of repeats, 17, is disregarded. At least one allele with 17 repeats was found in 40% of the DNA samples analysed. This pattern of distribution has been reported consistently across previous studies of populations with European ancestry. Mean repeat number in this cohort was 18.48. Having less than 15 repeats was a rare occurrence found in only 239/14758 alleles, as well as repeat numbers above the IA range, which as expected was very uncommon as it represents individuals at risk for clinical HD. Four individuals had alleles with extremely short *HTT* CAG repeats (equal or less than 3; one individual was homozygous). (Table 1;Fig. 3.)

**Table 1:** Distribution of allele sizes. In total, 14758 alleles were investigated from 7379 individuals.

Number (all alleles)	14758
Mean	18,48
Median	17,00
Std. Deviation	3,342
Range	3–42
Intermediate alleles	3,4%
Reduced penetrance alleles	0,2%
Full penetrance alleleles	0,01%

## Discussion

This represents the largest investigated cohort where the number of CAG repeats in the *HTT* gene is fully known, not selected for relationship to known expanded *HTT* gene carriers or HD patients.

This allows us to assess the true prevalence of IA, reduced penetrance alleles and also HD itself in an unbiased manner, something that is quite unique and hard to truly assess by finding patients in for instance hospital records. Reasons for this is the variable clinical phenotype of HD, where some patients may be misdiagnosed, or never come under medical attention at all.

Although two individuals found with full penetrance alleles does not reliably allow for calculating incidence, the result suggests a high prevalence (27/100,000) in this region of Sweden, in line with the prevalence (22.1/100,000) described in another northern Swedish county, Jämtland^20^.

Interestingly, four individuals with very short CAG repeats were found, a finding not reported before. This expands our understanding about the variation of CAG repeat length in the *HTT* gene in humans.

Further, this cohort contains enough individuals and matched controls to be able to investigate several important issues pertaining to the *HTT* gene expansion length. By linkage of data to the various registers available from the Swedish National Board of Health and Welfare and Statistics Sweden, we can find medical diagnoses (International Classification of Diseases (ICD) codes for each visit), causes of death, incidence of cancer, data concerning other medical issues as well as data concerning education, income, usage of social security systems and other data points indirectly influenced by health issues. By this method, we can in an unbiased manner investigate any influence the expansion length in the *HTT* gene may have on the risk of other diseases.

This is made possible by the fact that samples have been provided in NSHDS and can be linked to individuals by their Swedish social security number (a unique 10-digit personal identification number). This has of course raised ethical concerns, but steps have been taken to prevent the possibility of coupling allele data to individuals offering samples. The samples have been provided to the genotyping centre (Uppsala University) with only an identifying key pertaining to data from NSHDS, with the personal identification number and key remaining at Umeå University. Data from individual patients can be extracted from national registries by virtue of social security number via Umeå University and transferred to the data analysing centre coupled only to the identifying key.

Thus, subsequent analyses can be performed while the knowledge of genetic status in single individuals traceable via NSHDS remains unknown to persons able to identify them.

The main weakness is the fact that the cohort cannot be described as completely unbiased, since the individuals included in the cohort is linked to MI. We have addressed this and checked the occurrence of retrospective MI in IA carriers and controls, and no difference was seen. It should also be noted that participation in NSHDS of course is voluntary, and there might be a risk that persons with family history of HD are more probable than others to refrain from participation, thus possibly underestimating the true prevalence of expanded *HTT*-alleles. Since our findings are plausible and in accordance with previous prevalence studies in Sweden and neighbouring regions, this seems unlikely.

## Collaborations

Due to ethical constraints and data protection laws, data are not freely available. Pseudonymized data are stored at Uppsala University, while additional information including personal identification numbers are stored at the Biobank Research Unit, Umeå University. These sets can only be matched following new applications to the ethical review boards. Possibilities for collaborations remain open, including suggestions for further genetic characterization and should be addressed to the corresponding author and will be reviewed by the PI of the SHAPE cohort (JS) and the PI:s and Expert Committee of NSHDS.

## Supplementary information

Supplementary Information.

## References

[1] A novel gene containing a trinucleotide repeat that is expanded and unstable on Huntington’s disease chromosomes. The Huntington’s Disease Collaborative Research Group. *Cell*. **72**(6), 971–83, 10.1016/0092-8674(93)90585-e, [published Online First: 1993/03/26] (1993). 10.1016/0092-8674(93)90585-e 8458085

[2] Semaka, A. *et al*. Predictive testing for Huntington disease: interpretation and significance of intermediate alleles. *Clin Genet*. **70**(4), 283–94, 10.1111/j.1399-0004.2006.00668.x, [published Online First: 2006/09/13] (2006). 10.1111/j.1399-0004.2006.00668.x 16965319

[3] Corrochano, S. *et al*. A genetic modifier suggests that endurance exercise exacerbates Huntington’s disease. *Hum Mol Genet*. **27**(10), 1723-31, 10.1093/hmg/ddy077 [published Online First: 2018/03/07] (2018). 10.1093/hmg/ddy077 PMC5932560 29509900

[4] Garcia-Gorro, C. *et al*. An active cognitive lifestyle as a potential neuroprotective factor in Huntington’s disease. *Neuropsychologia*. **122**, 116–24, 10.1016/j.neuropsychologia.2018.10.017, [published Online First: 2018/12/20] (2019). 10.1016/j.neuropsychologia.2018.10.017 30563619

[5] Goold, R. *et al*. FAN1 modifies Huntington’s disease progression by stabilizing the expanded HTT CAG repeat. *Hum Mol Genet*. **28**(4), 650-61, 10.1093/hmg/ddy375, [published Online First: 2018/10/26] (2019). 10.1093/hmg/ddy375 PMC6360275 30358836

[6] Baig, S. S., Strong, M. & Quarrell, O. W. The global prevalence of Huntington’s disease: a systematic review and discussion. *Neurodegener Dis Manag*. **6**(4), 331-43, 10.2217/nmt-2016-0008, [published Online First: 2016/08/11] (2016). 10.2217/nmt-2016-0008 27507223

[7] Squitieri, F. & Jankovic, J. Huntington’s disease: how intermediate are intermediate repeat lengths? *Mov Disord*. **27**(14), 1714–7, 10.1002/mds.25172, [published Online First: 2012/09/26] (2012) 10.1002/mds.25172 23008174

[8] Jones, L., Houlden, H. & Tabrizi, S. J. DNA repair in the trinucleotide repeat disorders. *Lancet Neurol*. **16**(1), 88–96, 10.1016/S1474-4422(16)30350-7 [published Online First: 2016/12/17] (2017). 10.1016/S1474-4422(16)30350-7 27979358

[9] Kenney, C., Powell, S. & Jankovic, J. Autopsy-proven Huntington’s disease with 29 trinucleotide repeats. *Mov Disord*. **22**(1), 127–30, 10.1002/mds.21195, [published Online First: 2006/11/23] (2007). 10.1002/mds.21195 17115386

[10] Groen, J. L. *et al*. Late-onset Huntington disease with intermediate CAG repeats: true or false? *J Neurol Neurosurg Psychiatry*. **81**(2), 228–30, 10.1136/jnnp.2008.170902, [published Online First: 2010/02/11] (2010). 10.1136/jnnp.2008.170902 20145031

[11] Wright, G. E. B. *et al*. Length of uninterrupted CAG repeats, independent of polyglutamine size, results in increased somatic instability and hastened age of onset in Huntington disease. *bioRxiv*. 533414, 10.1101/533414 (2019). 10.1016/j.ajhg.2019.04.007 PMC6556907 31104771

[12] Cubo, E. *et al*. Clinical manifestations of intermediate allele carriers in Huntington disease. *Neurology*. **87**(6), 571–8, 10.1212/WNL.0000000000002944, [published Online First: 2016/07/13] (2016). 10.1212/WNL.0000000000002944 27402890

[13] Ha, A. D., Beck, C. A. & Jankovic, J. Intermediate CAG Repeats in Huntington’s Disease: Analysis of COHORT. *Tremor Other Hyperkinet Mov (N Y)*. **2**, 10.7916/D8FF3R2P, [published Online First: 2013/02/27] (2012). 10.7916/D8FF3R2P PMC3569951 23440000

[14] Killoran, A. *et al*. Characterization of the Huntington intermediate CAG repeat expansion phenotype in PHAROS. *Neurology*. **80**(22), 2022–7, 10.1212/WNL.0b013e318294b304, [published Online First: 2013/04/30] (2013). 10.1212/WNL.0b013e318294b304 PMC3716408 23624566

[15] Savitt, D. & Jankovic, J. Clinical phenotype in carriers of intermediate alleles in the huntingtin gene. *J Neurol Sci*. **402**, 57–61, 10.1016/j.jns.2019.05.010, [published Online First: 2019/05/20] (2019). 10.1016/j.jns.2019.05.010 31103960

[16] Gardiner, S. L. *et al*. Huntingtin gene repeat size variations affect risk of lifetime depression. *Transl Psychiatry*. **7**(12), 1277, 10.1038/s41398-017-0042-1, [published Online First: 2017/12/12] (2017). 10.1038/s41398-017-0042-1 PMC5802693 29225330

[17] Uhlmann, W. R. & Roberts, J. S. Ethical issues in neurogenetics. *Handb Clin Neurol*. **147**, 23–36, 10.1016/B978-0-444-63233-3.00003-8, [published Online First: 2018/01/13] (2018). 10.1016/B978-0-444-63233-3.00003-8 PMC5896012 29325614

[18] Kay, C. *et al*. The molecular epidemiology of Huntington disease is related to intermediate allele frequency and haplotype in the general population. *Am J Med Genet B Neuropsychiatr Genet*. **177**(3), 346–57, 10.1002/ajmg.b.32618, [published Online First: 2018/02/21] (2018). 10.1002/ajmg.b.32618 29460498

[19] Mattsson, B. Huntington’s chorea in Sweden. *Acta Psychiatr Scand Suppl*. **255**, 221–35, [published Online First: 1974/01/01] (1974). 4282554

[20] Roos, A. K., Wiklund, L & Laurell, K. Discrepancy in prevalence of Huntington’s disease in two Swedish regions. *Acta Neurol Scand*. **136**(5), 511–15, 10.1111/ane.12762, [published Online First: 2017/04/11] (2017). 10.1111/ane.12762 28393354

[21] Hallmans, G. *et al*. Cardiovascular disease and diabetes in the Northern Sweden Health and Disease Study Cohort - evaluation of risk factors and their interactions. *Scand J Public Health Suppl*. **61**, 18–24, 10.1080/14034950310001432, [published Online First: 2003/12/09] (2003). 10.1080/14034950310001432 14660243

[22] Norberg, M. *et al*. The Vasterbotten Intervention Programme: background, design and implications. *Glob Health Action*. **3**, 10.3402/gha.v3i0.4643, [published Online First: 2010/03/27] (2010). 10.3402/gha.v3i0.4643 PMC2844807 20339479

[23] Eriksson, M. *et al*. Large improvements in major cardiovascular risk factors in the population of northern Sweden: the MONICA study 1986–2009. *J Intern Med*. **269**(2), 219–31, 10.1111/j.1365-2796.2010.02312.x, [published Online First: 2010/12/17] (2011). 10.1111/j.1365-2796.2010.02312.x 21158982

[24] Stegmayr, B., Lundberg, V. & Asplund, K. The events registration and survey procedures in the Northern Sweden MONICA Project. *Scand J Public Health Suppl*. **61**, 9–17, 10.1080/14034950310001441, [published Online First: 2003/12/09] (2003). 10.1080/14034950310001441 14660242

[25] Gasser, T. Advances in the genetics of movement disorders: implications for molecular diagnosis. *J Neurol*. **244**(6), 341-8, 10.1007/s004150050100, [published Online First: 1997/06/01] (1997). 10.1007/s004150050100 9249618

[26] Apolinario, T. A., Paiva, C. L. & Agostinho, LA. REVIEW-ARTICLE Intermediate alleles of Huntington’s disease HTT gene in different populations worldwide: a systematic review. *Genet Mol Res*. **16**(2), 10.4238/gmr16029648, [published Online First: 2017/04/08] (2017). 10.4238/gmr16029648 28387881

[27] Kay, C., Hayden, M. R. & Leavitt, B. R. Epidemiology of Huntington disease. *Handb Clin Neurol*. **144**, 31–46, 10.1016/B978-0-12-801893-4.00003-1, [published Online First: 2017/09/28]. (2017). 10.1016/B978-0-12-801893-4.00003-1 28947124

