# Supplementary material for: High frequency of intermediary alleles in the HTT gene in Northern Sweden - The Swedish Huntingtin Alleles and Phenotype (SHAPE) study
Source: Sci Rep. 2020 Jun 17;10:9853. doi: 10.1038/s41598-020-66643-0 (PMC7299994; doi:10.1038/s41598-020-66643-0)
Supplement: Supplementary file 1 — Supplementary Information. [file 41598_2020_66643_MOESM1_ESM.docx]

Supplementary info

High frequency of intermediary alleles in the HTT gene in Northern Sweden - The Swedish Huntingtin Alleles and Phenotype (SHAPE) study

Jimmy Sundblom*^1^, Valter Niemelä^2^, Maria Ghazarian^2^, Ann-Sofi Strand^3^, Ingvar A. Bergdahl^4^, Jan-Håkan Jansson^5^, Stefan Söderberg^6^, Eva-Lena Stattin^7^

^1^ Dept. of Neuroscience, Neurosurgery; Uppsala University, Uppsala, Sweden

^2^ Dept. of Neuroscience, Neurology; Uppsala University, Uppsala, Sweden

^3^ Science for Life Laboratory (SciLifeLab), Department of Immunology, Genetics, and Pathology; Uppsala University, Uppsala, Sweden.

^4^ The Biobank Research Unit; Umeå University, Umeå, Sweden.

^5^ Department of Public Health and Clinical Medicine, Research Unit Skellefteå; Umeå University, Umeå, Sweden

^6^Department of Public Health and Clinical Medicine, Heart Centre, Umeå University, Umeå, Sweden.

^7^Department of Immunology, Genetics and Pathology, Science for Life Laboratory; Uppsala University, Uppsala, Sweden,

| grouping | variable Name | | categorizations |
| --- | --- | --- | --- |
| ID variables | **Set_FIA3** | | Fall and its controllers grouping numbers |
|  | **FIAnum_FIA3** | | This code is uniquely created for FIA3 study, used primarily as ID |
|  | **DNA_FIA** | | **Participation in FIA**  1 = FIA1  2 = FIA2  3 = FIA3  **Cross Match:**  = 12 involved in both FIA1 and 2  = 13 involved in both FIA1 and 3  = 23 involved in both FIA2 and 3  = 123 involved in both FIA1, 2 and 3 |
| Background variables | **sample_date** | | Blood Sampling Date  (year month day)  *Note: Please note that sample_date and q_date differ in some cases.* |
|  | **q_date** | | Date of survey  (year month day)  *Note: Please note that sample_date and q_date differ in some cases.* |
|  | **gender** | | Sex  M = Male  F = Female |
|  | **delproj** | | Delkohort  MA = Mammography Cohort (only included DNA_FIA)  MO = MONICA screening  VIP = Västerbotten Intervention Program |
|  | **fallkontroll_FIA3** | | Case F =  C = Control |
|  | **heartdat** | | Date of heart attack  Format: YYMMDD (year, month, day) |
|  | **Age** | | Age at the time of sampling |
|  | **fasta_enk**  **Applies only to persons who have participated in the VIP** | | Solid State - comes to blood sugar, cholesterol and triglycerides downloaded from the survey, are only mentioned in optical surveys in VIP.  1 = 8 hours or more  2 = less than 8 hours  *Note: There fasta_enk not found information about the status of the variable fasta_prov.*  *Fasta_enk is connected to q_date and fasta_prov is connected to sample_date (which differ in some cases).* |
|  | **fasta_prov** | | Solid State - apply the saved research samples, as well as blood sugar, cholesterol and triglycerides on the value of the variable fasta_enk missing.  0 = 0-4 hour fast  1 = 4-6 hours fasting  2 = 6-8 hour fast  3 => 8-hour fast  *Note: Fasta_prov is connected to sample_date and fasta_enk is connected to q_date (which differ in some cases).* |
|  | **info** | | Indicates various information about the participant |
|  | **uppfyller_FIA1o2_kriterier** | | 1 = Yes  2 = No  3 = Applies for the previously engaged in FIA1 and FIA2:  Met the criteria for imposing the FIA1 and 2 but not anymore due date records |
|  | **prosp** | | 0 = Retrospective  1 = Prospective  Indicates if halyard dates of sample / data is present (prospectively) or after (retrospectively) exact diagnosis of heart attack. In Enkät_FIA3 included only prospective cases. |
|  | **tidigare_hjartinfarkt**  (The variable applies only new cases in FIA3, that is not the case previously included in FIA1 or 2). | | 1 = First time infarction, myocardial infarction no prior medical examination  2 = Heart attacks before and after the medical examination  3 = Heart attack just before health assessment |
|  | **plotslig_dod**  (The variable applies only new cases in FIA3, that is not the case previously included in FIA1 or 2). | | 1 = Death within <1 hour  24 = Death within 1-24 hours + likely death in <24 hours |
| Medical and anthropometricvariables | **LENGTH** | **Body length in cm**  *Note: If the variable "l_v_uppskattad" is missing, the value is measured in relation to sampling. If "l_v_uppskattad" = 1, the value itself appreciated.* | |
|  | **weight** | **Weight in kg**  *Note: If the variable "l_v_uppskattad" is missing, the value is measured in relation to sampling. If "l_v_uppskattad" = 1, the value itself appreciated.* | |
|  | **l_v_uppskattad** | 1 = self estimated length / weight | |
|  | **BMI** | **The body mass index** - weight in kg / (height m) 2  (Calculated from height and weight, whether it's self-estimated or measured value.) | |
|  | **waist** | **Number cm around the waist** | |
|  | **Total cholesterol at the time of sampling** | | |
|  | **skol_mo**  Applies only to participants of MONICA | **Total cholesterol mmol / l.**  Analysis of Laboratory Medicine, NUS. | |
|  | **school**  Applies only to participants in the VIP | **Total cholesterol mmol / l.**  Analysis Reflotron before 2009-09-01, 2009-09-01 from the analysis of Laboratory Medicine, NUS.  If a breakdown of the variable needs to be done is the variable "efter_090901" to help where 0 = analysis Reflotron, ie sampled 2009-09-01 and earlier and 1 = analysis kemlab, that samples taken after 2009-09-01. | |
|  | **HDL** | **HDL cholesterol mmol / l** | |
|  | **LDL**  Applies only to participants in the VIP | **Ldl cholesterol mmol / l** | |
|  | **Serum triglycerides at the time of sampling** | | |
|  | **stg_mo**  Applies only to participants of MONICA | **Triglycerides mmol / l.**  Analysis of Laboratory Medicine, NUS. | |
|  | **stg**  Applies only to participants in the VIP | **Triglycerides mmol / l.**  Analysis Reflotron before 2009-09-01, 2009-09-01 from the analysis of Laboratory Medicine, NUS.  If a breakdown of the variable needs to be done is the variable "efter_090901" to help where 0 = analysis Reflotron, ie sampled 2009-09-01 and earlier and 1 = analysis kemlab, that samples taken after 2009-09-01. | |
|  | If a comparison to be made between the different analytical methods are available to the following formula developed by VHU Group:  **Applies only to participants in the VIP**  EBF recommends that the applicant carefully evaluate differences between measured and adjusted values ​​of the lowest and highest percentiles. | | |
|  | TG = Triglyceride  Chol = Cholestrol   \| S-TG - Reflotron \| 0.177 + (0.932 × S-TG - Clin Chemistry) \| \| --- \| --- \| \| S-Chol - Reflotron \| 0.170 + (0.939 × S-Chol - Clin Chemistry) \| \|  \| \| \| S-TG Clin Chemistry \| 0.888 + (0.139 × S-TG - Reflotron) \| \| S-Chol - Clin Chemistry \| 0.738 + (0901 × S-Chol - Reflotron) \| | | |
|  | **Blood glucose at the time of sampling** | | |
|  | **blods0** | **Glucose 0-hour value**  (Oral glucose tolerance test)  VIP: Capillary plasma glucose through the years. (Reflotron / Hemocue)  MO: venous plasma glucose through the years. Analysis of Laboratory Medicine, NUS until 2004, then analyzed by HemoCue. | |
|  | **blods2** | **Blood glucose 2-hour value**  (Oral glucose tolerance test)  VIP: Capillary plasma glucose through the years.  (Reflotron / Hemocue)  MO: venous plasma glucose through the years. Analysis of Laboratory Medicine, NUS until 2004, then analyzed by HemoCue. | |
|  | **Blood pressure at the time of sampling** | | |
|  | **btsyst**  Applies only to participants of MONICA | **Systolic blood pressure.**  Captured seated. | |
|  | **btdiast**  Applies only to participants of MONICA | **Diastolic blood pressure.**  Captured seated. | |
|  | **SBT**  Applies only to participants in the VIP | **Systolic blood pressure.**  Measured in supine position before 2009-09-01 and from 2009-09-01, measured in a sitting position.  If a breakdown of the variable needs to be done is the variable "efter_090901" to help where 0 = lying blood pressure, ie blood pressure taken 2009-09-01 and earlier and 1 = sitting blood pressure, ie blood pressure taken after 2009-09-01. | |
|  | **DBT**  Applies only to participants in the VIP | **Diastolic blood pressure.**  Measured in supine position before 2009-09-01 and from 2009-09-01, measured in a sitting position.  If a breakdown of the variable needs to be done is the variable "efter_090901" to help where 0 = lying blood pressure, ie blood pressure taken 2009-09-01 and earlier and 1 = sitting blood pressure, ie blood pressure taken after 2009-09-01. | |
|  | **efter_090901**  Applies only to participants in the VIP | 0 = sample taken 2009-09-01 Earlier  1 = sample taken after 2009-09-01 | |
|  | **In case comparison between lying and sitting blood pressure is below algorithm developed by VHU group.**  *Note that the algorithm only applies to 40-, 50- and 60-year-olds in the VIP.* | | |
|  | **Conversion Formulas for sitting and lying in blood pressure VHU**   \| 40 years \| men \| Sitting systolic BP \| 21612 + (0.835 x lying syst BT) \| \| --- \| --- \| --- \| --- \| \|  \|  \| Landscape systolic BP \| 24595 + (0.792 x Sit syst BT) \| \|  \|  \|  \|  \| \|  \|  \| Seated diastolic BP \| 14463 + (0.848 x lying diast BT) \| \|  \|  \| Landscape diastolic BP \| 17282 + (0.753 x Sit diast BT) \| \|  \|  \|  \|  \| \|  \| Women \| Sitting systolic BP \| 19922 + (0.830 x lying syst BT) \| \|  \|  \| Landscape systolic BP \| 8669 + (0919 x Sit syst BT) \| \|  \|  \|  \|  \| \|  \|  \| Seated diastolic BP \| 13680 + (0.847 x lying diast BT) \| \|  \|  \| Landscape diastolic BP \| 5784 + (0.890 x Sit diast BT) \| \|  \|  \|  \|  \| \| 50 year \| men \| Sitting systolic BP \| 19 748 + (0861 x lying syst BT) \| \|  \|  \| Landscape systolic BP \| 9.850 + (0.910 x Sit syst BT) \| \|  \|  \|  \|  \| \|  \|  \| Seated diastolic BP \| 13390 + (0.878 x lying diast BT) \| \|  \|  \| Landscape diastolic BP \| 12 363 + (0812 x Sit diast BT) \| \|  \|  \|  \|  \| \|  \| Women \| Sitting systolic BP \| 12,723 + (0.906 x lying syst BT) \| \|  \|  \| Landscape systolic BP \| 16051 + (0.859 x Sit syst BT) \| \|  \|  \|  \|  \| \|  \|  \| Seated diastolic BP \| 17675 + (0.800 x lying diast BT) \| \|  \|  \| Landscape diastolic BP \| 13566 + (0.798 x Sit diast BT) \| \|  \|  \|  \|  \| \| 60 years \| men \| Sitting systolic BP \| 20 246 + (0853 x lying syst BT) \| \|  \|  \| Landscape systolic BP \| 7763 + (0.936 x Sit syst BT) \| \|  \|  \|  \|  \| \|  \|  \| Seated diastolic BP \| 16308 + (0.833 x lying diast BT) \| \|  \|  \| Landscape diastolic BP \| 9029 + (0.864 x Sit diast BT) \| \|  \|  \|  \|  \| \|  \| Women \| Sitting systolic BP \| 13817 + (0.900 x lying syst BT) \| \|  \|  \| Landscape systolic BP \| 9.999 + (0.914 x Sit syst BT) \| \|  \|  \|  \|  \| \|  \|  \| Seated diastolic BP \| 15084 + (0.836 x lying diast BT) \| \|  \|  \| Landscape diastolic BP \| 7992 + (0.870 x Sit diast BT) \| | | |
| survey Variables | **civil**  VIP: 1985 ->  MONICA: 1986 - 2014 | **Marital status?** 1 = married  2 = Married / cohabitant + remarried / re Attached  3 = divorced / separated  4 = Widow / er  Double digits or greater indicates what options combined. | |
|  | **training**  VIP: 1985 ->  MONICA: 1986 - 2014 | **Education?** 1 = Folkskola + 9 year old primary (mandatory school)  2 = Folkhögskola equivalent. grundsk. + Real + girls school + professional / skilled school  3 = Folkhögskola equivalent. gymnasium + secondary / girls school equivalent. secondary competence  4 = Academic training / college  5 = Do not finished elementary school or other basic training (only MONICA 2014)  6 = High school, elementary school, training or equivalent (not more than 9 years)  (Only MONICA 2014) | |
|  | **partner**  VIP: 1985 ->  MONICA: 1986 - 2014 | **Who cohabits with you?** 1 = Only an adult (spouse or partner)  2 = Only children  3 = Adult and children  4 = Other / Other  5 = Living alone  6 = Adults with or without children (only MONICA)  *Note: In a number of surveys are the "not living alone," these have been coded as 9999th* | |
|  | **shift work**  VIP: 1985 ->  MONICA: 1986 - 2009 | **If you have shift work / weekend service?** 1 = Yes  2 = No | |
|  | **sick**  VIP: 1989 ->  Applies only to participants in the VIP | **Have you been on sick leave for more than six months?** 1 = Yes 2 = No | |
|  | **arbsjukpens_8604**  MONICA: 1986-2004 Applies only to participants of MONICA 1986-2004 | **Is currently on sick leave or retirement / early retirement / sjukpens / contributions?** 1 = Yes, pension / contribution  2 = Yes, sick  3 = No | |
|  | **arbsjukpens_0409**  MONICA: 2004-2009 Applies only to participants of MONICA 2004-2009 | **Right now on sick leave, the old-age, disability, disability pension or temporary disability?** 1 = Yes, retirement / disability pension, full time  2 = Yes, retirement / disability pension, part-time 3 = Yes, early / temporary disability 4 = Yes, sick  5 = No | |
|  | **arbsjukpens_14**  MONICA: 2014  Applies only to participants of MONICA 2014 | **Are you currently one of the following?** 1 = Yes, age pensioner  2 = Yes, contracts retired  3 = Yes early retirement, on disability  4 = Yes, long-term sick  5 = No  Double digits or greater indicates what options combined. | |
|  | **arbyrke_8699**  MONICA: 1986-1999 Applies only to participants of MONICA 1986-1999 | **Occupation Classification (NYK)**If interested in this variable, please contact Robert Lundqvist for more details. *Robert.Lundqvist*[@ nll.se](mailto:per.ivarsson@medicin.umu.se) | |
|  | **arbyrke_0409**  MONICA: 2004-2009 Applies only to participants of MONICA 2004-2009 | **Occupation Classification (NYK)**If interested in this variable, please contact Robert Lundqvist for more details. *Robert.Lundqvist*[@ nll.se](mailto:per.ivarsson@medicin.umu.se) | |
|  | **ansttyp_a - ansttyp_m**  VIP: 1985 ->  MONICA: 1986-2014 | **Employee?** **ansttyp_a** = Fast employment  **ansttyp_b** = Temporary employment, temporary, emergency work, labor action  **ansttyp_c** = Work at home  **ansttyp_d** = unemployed  **ansttyp_e** = Studying  **ansttyp_f** = Self employed  **ansttyp_g** = Disability / sickness pension, FB (only VIP)  **ansttyp_h** = Disability / sickness pension, partial retirement (only VIP)  **ansttyp_i**= Disability / sickness pension, unspec. whole or partial retirement  **ansttyp_j** = Other, other (only MONICA)  **ansttyp_k** = Absence or parental leave (only MONICA 2014)  **ansttyp_l** = Senior age retirement, retired agreement (only MONICA)  **ansttyp_m** = Long-term sick (only MONICA 2014) | |
|  | **country of origin**  VIP: 1985 ->  MONICA: 1986-2014 | **What country are you from?** 1 = Sweden  2 = Other country, what? (See ursprungsland_vilket) | |
|  | **ursprungsland_vilket**  VIP: 1985 ->  MONICA: 1986-2014 | **Defines which country the person comes from** | |
|  | **halsojf**  VIP: 1985 -2003  MONICA: 1990-2014 | **General health compared with others of your age?** 1 = Better  2 = About the same  3 = Worse | |
|  | **halsoal**  VIP: 1985-1994  MONICA: 1990-1994 | **How would you rate your overall health?** 1 = Good  2 = neither bad nor good / something therebetween  3 = poor | |
|  | **halsoar**  VIP: 1989 ->  MONICA: 1986, 1999 to 2014 | **Health status last year?** 1 = Poor  2 = Somewhat bad  3 = Reasonable  4 = Fairly good  5 = Very good | |
|  | **hjartinf_foraldrar_syskon**  VIP: 1989 ->  Applies only to participants in the VIP | **Have any of your parents or siblings before 60 years of age suffered a heart attack or stroke / clot in the brain?** 1 = Yes  2 = No  3 = DK | |
|  | **mislakt_9409**  MONICA: 1994-2009 Applies only to participants of MONICA 1994-2009 | **Someone in the family who had died of a heart attack before age 65?** 1 = Yes  2 = No  3 = DK | |
|  | **mifamilj_14**  MONICA: 2014 Applies only to participants of MONICA 2014 | **Someone in your family who have fallen ill / had died of a heart attack?** 1 = Yes  2 = No  3 = DK | |
|  | **cvsslakt_9409**  MONICA: 1994-2009 Applies only to participants of MONICA 1994-2009 | **Is there anyone in your family who died of a stroke (clot or bleeding in the brain) before age 65?** 1 = Yes  2 = No 3 = DK | |
|  | **cvsfamilj_14**  MONICA: 2014 Applies only to participants of MONICA 2014 | **Someone in your family who are ill / dies of stroke / stroke?** 1 = Yes  2 = No 3 = DK | |
|  | **diab_foraldrar_syskon**  VIP: 1989 ->  MONICA: 1999-2014 | **Occurs diabetes in either of your parents and siblings?** 1 = Yes  2 = No  3 = DK | |
|  | **beskbltr**  VIP: 1988 ->  MONICA: 1986-2014 | **If at any point you been told that you have high blood pressure?**  1 = Yes  2 = No | |
|  | **drugs**  (Med_C5a-med_C5f, smartmed, med_acehjar, med_acecvs, andra_mediciner)  VIP: 1985 ->  MONICA: 1986-2014 | **Have you during the past 14-day period used any of the following medications?** Blood Pressure Medicine, med_C5a  1 = yes (VIP's only option 1)  2 = no  3 = Uncertain  Heart / angina medicine, med_C5b (only VIP)  1 = yes  Sedative hypnotics, med_C5c (only VIP)  1 = yes  Ulcer / gastritis medicine, med_C5d (only VIP)  1 = yes Lipid lowering, med_C5e 1 = yes (VIP's only option 1)  2 = no  3 = DK No, do not use any of the above medications, med_C5f (Only VIP) 1 = yes Analgesics, smartmed (Only VIP + MONICA 1986) 1 = yes  2 = no (Only MONICA 1986)  Aspirin for cardiovascular disease, med_asahjar (only MONICA)  1 = yes  2 = For another sjd  3 = no  Aspirin against stroke, med_asacvs (only MONICA)  1 = yes  2 = For another sjd  3 = no | |
|  |  | Have you during the past 14-day period, any other medicines prescribed by a doctor, such as antidepressants, epilepsy, penicillin or hormones, or any medicine that is not prescribed by a doctor, such as aspirin, vitamin or iron preparations, omega -3, or other dietary supplements, natural medicine or other? - andra_mediciner  1 = yes  2 = no | |
|  | **from1-med29**  VIP: 1985 ->  MONICA: 1986-2014 | **ATC code** | |
|  | **Diabet**  VIP: 1986 ->  MONICA: 1986-2014 | **Do you have diabetes?** 1 = Yes  2 = No  3 = DK (only MONICA 2014) | |
|  | **diabetesbehandling_a - diabetesbehandling_d**  VIP: 1988 ->  MONICA: 1986-2014 | **Treat you with ...** **diabetesbehandling_a** = Diet and exercise alone  **diabetesbehandling_b** = Tablets  **diabetesbehandling_c** = Insulin  **diabetesbehandling_d** = No treatment with the above | |
|  | **gestational diabetes**  VIP: 2011 ->  MONICA: 1999-2014 | **Have you had gestational diabetes?** 1 = Yes  2 = No  3 = Has no children (only MONICA)  4 = DK (only MONICA) | |
|  | **infarkt_sjukhus**  VIP: 1989 ->  MONICA: 1986-2014 | ***Have you been in the hospital for certain heart attack?***  1 = Yes  2 = No  3 = Do not know if safe (only MONICA) | |
|  | **infarkt_sjukhus_ar**  VIP: 1991 ->  MONICA: 1986-2014 | ***If you been in the hospital for certain heart attack, what year?*** | |
|  | **SF36_RAND_1**  VIP: 2003 ->  Applies only to participants in the VIP | ***In general, would you say your health is:***  1 = Excellent  2 = Very good  3 = Good  4 = somewhat  5 = Poor | |
|  | **SF36_RAND _2**  VIP: 2003 ->  Applies only to participants in the VIP | ***Compared with a year ago, how would you assess your overall health now?***  1 = Much better now than one year ago  2 = Somewhat better now than one year ago  3 = About the same  4 = Somewhat worse now than one year ago  5 = Much worse now than one year ago | |
|  | **SF36_RAND _3a**  VIP: 2003 ->  Applies only to participants in the VIP | ***Are you due to your physical health is limited in strenuous activities such as running, lifting heavy objects, participating in strenuous sports?***  1 = Yes, very limited  2 = Yes, a bit limited  3 = No, not at all limited | |
|  | **SF36_RAND _3b**  VIP: 2003 ->  Applies only to participants in the VIP | ***Are you due to your physical health limited at moderately strenuous activities, such as moving a table, vacuuming, forest walks or gardening?***  1 = Yes, very limited  2 = Yes, a bit limited  3 = No, not at all limited | |
|  | **SF36_RAND _3c**  VIP: 2003 ->  Applies only to participants in the VIP | ***Are you due to your physical health limited at moderately strenuous activities, such as lifting or carrying groceries?***  1 = Yes, very limited  2 = Yes, a bit limited  3 = No, not at all limited | |
|  | **SF36_RAND _3D**  VIP: 2003 ->  Applies only to participants in the VIP | ***Are you due to your physical health limited at moderately strenuous activity, such as walking up several flights of stairs?***  1 = Yes, very limited  2 = Yes, a bit limited  3 = No, not at all limited | |
|  | **SF36_RAND _3e**  VIP: 2003 ->  Applies only to participants in the VIP | ***Are you due to your physical health limited at moderately strenuous activity, such as walking up a flight of stairs?***  1 = Yes, very limited  2 = Yes, a bit limited  3 = No, not at all limited | |
|  | **SF36_RAND _3f**  VIP: 2003 ->  Applies only to participants in the VIP | ***Are you due to your physical health limited at moderately strenuous activities, such as bend or kneel down?***  1 = Yes, very limited  2 = Yes, a bit limited  3 = No, not at all limited | |
|  | **SF36_RAND _3g**  VIP: 2003 ->  Applies only to participants in the VIP | ***Are you due to your physical health limited at moderately strenuous activity, such as walking more than two kilometers?***  1 = Yes, very limited  2 = Yes, a bit limited  3 = No, not at all limited | |
|  | **SF36_RAND _3h**  VIP: 2003 ->  Applies only to participants in the VIP | ***Are you due to your physical health limited at moderately strenuous activity, such as walking a few hundred meters?***  1 = Yes, very limited  2 = Yes, a bit limited  3 = No, not at all limited | |
|  | **SF36_RAND _3i**  VIP: 2003 ->  Applies only to participants in the VIP | ***Are you due to your physical health limited at moderately strenuous activity, such as walking a hundred meters?***  1 = Yes, very limited  2 = Yes, a bit limited  3 = No, not at all limited | |
|  | **SF36_RAND _3j**  VIP: 2003 ->  Applies only to participants in the VIP | ***Are you due to your physical health limited at moderately strenuous activities, such as bathing or dressing yourself?***  1 = Yes, very limited  2 = Yes, a bit limited  3 = No, not at all limited | |
|  | **SF36_RAND _4a**  VIP: 2003 ->  Applies only to participants in the VIP | ***During the last four weeks, have you as a result of your physical health slashed the time you normally spend on work or other activities?***  1 = Yes  2 = No | |
|  | **SF36_RAND _4b**  VIP: 2003 ->  Applies only to participants in the VIP | ***During the last four weeks, have you as a result of your physical health Accomplished less than you would like?***  1 = Yes  2 = No | |
|  | **SF36_RAND _4c**  VIP: 2003 ->  Applies only to participants in the VIP | ***During the last four weeks, have you as a result of your physical health has been prevented from performing certain tasks or other activities?***  1 = Yes  2 = No | |
|  | **SF36_RAND _4d**  VIP: 2003 ->  Applies only to participants in the VIP | ***During the last four weeks, have you as a result of your physical health had difficulty performing work or other activities?***  1 = Yes  2 = No | |
|  | **SF36_RAND _5a**  VIP: 2003 ->  Applies only to participants in the VIP | ***During the last four weeks, have you as a result of emotional problems cut down the time you normally spent on work or other activities?***  1 = Yes  2 = No | |
|  | **SF36_RAND _5b**  VIP: 2003 ->  Applies only to participants in the VIP | ***During the last four weeks, have you as a result of emotional problems Accomplished less than you would have liked?***  1 = Yes  2 = No | |
|  | **SF36_RAND _5c**  VIP: 2003 ->  Applies only to participants in the VIP | ***During the last four weeks, have you as a result of emotional problems does not work or other activities as carefully as usual?***  1 = Yes  2 = No | |
|  | **SF36_RAND _6**  VIP: 2003 ->  Applies only to participants in the VIP | ***During the last four weeks, to what extent is there physical health or emotional problems interfered with your normal social activities with family, friends, neighbors or others?***  1 = not at all  2 = Little  3 = Moderately  4 = Very  5 = Very much | |
|  | **SF36_RAND _7**  VIP: 2003 ->  Applies only to participants in the VIP | ***How much bodily pain have you had during the past four weeks?***  1 = No  2 = Very easy  3 = Slight  4 = moderate  5 = Severe  6 = Very severe | |
|  | **SF36_RAND _8**  VIP: 2003 ->  Applies only to participants in the VIP | ***Over the past four weeks, how much did pain interfere with your normal work?***  1 = not at all  2 = Little  3 = Moderately  4 = Very  5 = Very much | |
|  | **SF36_RAND _9a**  VIP: 2003 ->  Applies only to participants in the VIP | ***How much of the time in the past four weeks have you felt really fit and strong?***  1 = All time  2 = Most of the time  3 = A lot of time  4 = Some of the time  5 = little of the time  6 = None of the time | |
|  | **SF36_RAND _9b**  VIP: 2003 ->  Applies only to participants in the VIP | ***How much of the time in the past four weeks have you felt very nervous?***  1 = All time  2 = Most of the time  3 = A lot of time  4 = Some of the time  5 = little of the time  6 = None of the time | |
|  | **SF36_RAND _9c**  VIP: 2003 ->  Applies only to participants in the VIP | ***How much of the time in the past four weeks have you felt so sad that nothing could cheer you up?***  1 = All time  2 = Most of the time  3 = A lot of time  4 = Some of the time  5 = little of the time  6 = None of the time | |
|  | **SF36_RAND _9d**  VIP: 2003 ->  Applies only to participants in the VIP | ***How much of the time in the past four weeks have you felt calm and peaceful?***  1 = All time  2 = Most of the time  3 = A lot of time  4 = Some of the time  5 = little of the time  6 = None of the time | |
|  | **SF36_RAND _9e**  VIP: 2003 ->  Applies only to participants in the VIP | ***How much of the time in the past four weeks have you been full of energy?***  1 = All time  2 = Most of the time  3 = A lot of time  4 = Some of the time  5 = little of the time  6 = None of the time | |
|  | **SF36_RAND _9f**  VIP: 2003 ->  Applies only to participants in the VIP | ***How much of the time in the past four weeks have you felt gloomy and sad?***  1 = All time  2 = Most of the time  3 = A lot of time  4 = Some of the time  5 = little of the time  6 = None of the time | |
|  | **SF36_RAND _9g**  VIP: 2003 ->  Applies only to participants in the VIP | ***How much of the time in the past four weeks have you felt worn out?***  1 = All time  2 = Most of the time  3 = A lot of time  4 = Some of the time  5 = little of the time  6 = None of the time | |
|  | **SF36_RAND _9h**  VIP: 2003 ->  Applies only to participants in the VIP | ***How much of the time in the past four weeks have you felt happy and happy?***  1 = All time  2 = Most of the time  3 = A lot of time  4 = Some of the time  5 = little of the time  6 = None of the time | |
|  | **SF36_RAND _9i**  VIP: 2003 ->  Applies only to participants in the VIP | ***How much of the time in the past four weeks have you felt tired?***  1 = All time  2 = Most of the time  3 = A lot of time  4 = Some of the time  5 = little of the time  6 = None of the time | |
|  | **SF36_RAND _10**  VIP: 2003 ->  Applies only to participants in the VIP | ***Over the past four weeks, how much of the time has your physical health or emotional problems interfered with your ability to socialize (eg health, family, friends, etc.)?***  1 = All time  2 = Most of the time  3 = Some of the time  4 = little of the time  5 = None of the time | |
|  | **SF36_RAND _11a**  VIP: 2003 ->  Applies only to participants in the VIP | ***I seem to have a little bit easier to get sick than others***  1 = just Compliant  2 = agree somewhat  3 = Uncertain  4 = Does not very good  5 = disagree | |
|  | **SF36_RAND _11b**  VIP: 2003 ->  Applies only to participants in the VIP | ***I'm as healthy as anyone of them I know***  1 = just Compliant  2 = agree somewhat  3 = Uncertain  4 = Does not very good  5 = disagree | |
|  | **SF36_RAND _11c**  VIP: 2003 ->  Applies only to participants in the VIP | ***I think my health will get worse***  1 = just Compliant  2 = agree somewhat  3 = Uncertain  4 = Does not very good  5 = disagree | |
|  | **SF36_RAND _11d**  VIP: 2003 ->  Applies only to participants in the VIP | ***My health is excellent***  1 = just Compliant  2 = agree somewhat  3 = Uncertain  4 = Does not very good  5 = disagree | |
|  | **livskvalitet_d1 - livskvalitet_d17**  VIP: 1996->  MONICA: 2004-2009 | Highlight how satisfied you are with your situation in various respects.  Very bad = 1st . . . really excellent = 7  **livskvalitet_d1** = Home and family situation  **livskvalitet_d2** = Living  **livskvalitet_d3** = Work situation  **livskvalitet_d4** = Economy  **livskvalitet_d5** = Leisure  You may experience some changes in himself over  the years, attempts to below highlight how you feel now.  Very bad = 1st . . . really excellent = 7  **livskvalitet_d6** Hearing =  **livskvalitet_d7** = sight  **livskvalitet_d8** = memory  **livskvalitet_d9** = Fitness  **livskvalitet_d10** = appetite  **livskvalitet_d11** = mood  **livskvalitet_d12** = energy  **livskvalitet_d13** = Patience  **livskvalitet_d14** = Confidence  **livskvalitet_d15** = Sleep  Do you feel important and appreciated  Very bad = 1st . . . really excellent = 7  **livskvalitet_d16** = Outside the home?  **livskvalitet_d17** = In the home? | |
|  | **ISSI**  Applies only to participants in the VIP | **Interview Schedule for Social Interaction (ISSI)** consists of a series of questions about one's social networks are added up to the scales ISSI_AVSI - Availability of Social Interaction (Social network size and functionality), and ISSI_AVAT - Availability of Attachment (Access to proximity and emotional support).  The index is based on 13 questions, these are socont-sochelp. These are assigned a score from 0-2 based on how many answer a question.  Questions related to AVSI and AVAT summed into a score that may have values ​​between 0-14 for AVSI score and 0-12 for AVAT score. | |
|  | **sockont**  VIP: 1985->  MONICA: 1986-2009 | **How many people do you know and have contact with the same interests as you?**  1 = No  2 = 1-2 people  3 = 3-5 people  4 = 6-10 people  5 = 11-15 persons  6 => 15 people | |
|  | **SOCSAM**  VIP: 1985->  MONICA: 1986-2009 | ***How many people, you know, meet or talk with you during a normal week?***  1 = No  2 = 1-2 people  3 = 3-5 people  4 = 6-10 people  5 = 11-15 persons  6 = more than 15 people | |
|  | **soclago**  VIP: 1985->  MONICA: 1986-2009 | **Is that about right many people you meet in your daily life? Would you like to see more or fewer people?**  1 = Fewer  2 = Occasionally many  3 = More | |
|  | **sochem**  VIP: 1985->  MONICA: 1986-2009 | ***How many friends do you have who can come to your home at any time and feel at home?***  (You would not worry about if it was untidy or if you were about to eat. Close relatives are not counted.)  1 = No  2 = 1-2 people  3 = 3-5 people  4 = 6-10 people  5 = 11-15 persons  6 = more than 15 people | |
|  | **soctala**  VIP: 1985->  MONICA: 1986-2009 | **How many are there that you can talk openly without thinking you?**  1 = No  2 = 1-2 people  3 = 3-5 people  4 = 6-10 people  5 = 11-15 persons  6 => 15 people | |
|  | **socstod**  VIP: 1985->  MONICA: 1986-2009 | **Is there a particular person to really get support from?** 1 = Yes  2 = Yes, but I do not  3 = No | |
|  | **socnara**  VIP: 1985->  MONICA: 1986-2009 | ***Is there anyone in particular who feel standing very close to you?***  1 = Yes  2 = Not sure  3 = No | |
|  | **soclyck**  VIP: 1985->  MONICA: 1986-2009 | ***Do you have anyone in particular that you can share your innermost feelings when you feel happy? Someone like myself feel happy just because you are there?***  1 = Yes  2 = No | |
|  | **socanfo**  VIP: 1985->  MONICA: 1986-2009 | **Do you have someone to share your innermost feelings with and confide in?**  1 = Yes  2 = No | |
|  | **soctrost**  VIP: 1985->  MONICA: 1986-2009 | ***It happens that someone holds about you to comfort and support?***  1 = Yes  2 = No | |
|  | **socupps**  VIP: 1985->  MONICA: 1986-2009 | **Do you think the home, or others appreciate what you do?**  1 = Yes  2 = Not enough  3 = No, not at all | |
|  | **soclana**  VIP: 1985->  MONICA: 1986-2009 | ***Are there people in your environment that is easy to ask for things, such as people that you know so well that you can borrow tools or kitchen stuff?***  1 = Yes  2 = No | |
|  | **sochelp**  VIP: 1985->  MONICA: 1986-2009 | **Apart from those at home, you can turn to someone when you are in trouble?**  1 = Yes  2 = No | |
|  | **socdelta**  VIP: 1989->  MONICA: 2004-2009 | ***Have you over the past year participated in any association, NGO, etc. with others (eg sports, study circle, theater, choir, political association)?***  1 = Yes  2 = No | |
|  | **socofta**  VIP: 1985->  MONICA: 2004-2009 | ***How often are you involved in association activities, clubs, study circles, etc. with others?***  1 = 1-2 times per year  2 = 1-2 times per month  3 = 1-2 times per week  4 = everyday  5 = DK | |
|  | **socforening_a - socforening_e**  VIP: 2001->  MONICA: 2004-2009 | *What is / are club activities you participate in?*  **socforening_a** = Sports & Exercise  **socforening-B** = Studies Group  **socforening_C** = theater  **socforening_d** = Choir  **socforening_e** = Other compound | |
|  | **arbfys**  VIP: 1986->  MONICA: 1986-2009 | **Is your work physically demanding?** 1 = Yes often  Yes sometimes = 2  3 = No seldom  4 = No almost never | |
|  | **arbfort**  VIP: 1985->  MONICA: 1986-2009 | **Does your job require you to work very fast?** 1 = Yes often  Yes sometimes = 2  3 = No seldom  4 = No almost never | |
|  | **arbpsyk**  VIP: 1986->  MONICA: 1986-2009 | **Is your work mentally strenuous?** 1 = Yes often  Yes sometimes = 2  3 = No seldom  4 = No almost never | |
|  | **arbhin**  VIP: 1985->  MONICA: 1986-2009 | **Do you have enough time for your work?** 1 = Yes often  Yes sometimes = 2  3 = No seldom  4 = No almost never | |
|  | **arbkrav**  VIP: 1985->  MONICA: 1986-2009 | **Are there conflicting demands of your work?** 1 = Yes often  Yes sometimes = 2  3 = No seldom  4 = No almost never | |
|  | **arbnytt**  VIP: 1985->  MONICA: 1986-2009 | **You will learn new things in your work?** 1 = Yes often  Yes sometimes = 2  3 = No seldom  4 = No almost never | |
|  | **arbski**  VIP: 1985->  MONICA: 1986-2009 | **Does your job skills?** 1 = Yes often  Yes sometimes = 2  3 = No seldom  4 = No almost never | |
|  | **arbide**  VIP: 1985->  MONICA: 1986-2009 | **Does your work require ingenuity?** 1 = Yes often  Yes sometimes = 2  3 = No seldom  4 = No almost never | |
|  | **arbrut**  VIP: 1985->  MONICA: 1986-2009 | **Does your work that you are doing the same things over and over again?** 1 = Yes often  Yes sometimes = 2  3 = No seldom  4 = No almost never | |
|  | **arbhur**  VIP: 1985->  MONICA: 1986-2009 | **Freedom to decide how the work should be done?** 1 = Yes often  Yes sometimes = 2  3 = No seldom  4 = No almost never | |
|  | **arbvad**  VIP: 1985->  MONICA: 1986-2009 | **Freedom to decide what should be done?** 1 = Yes often  Yes sometimes = 2  3 = No seldom  4 = No almost never | |
|  | **arbtala**  VIP: 1991->  MONICA: 1990-2009 | ***Do you usually able to talk to your colleagues during the break, if you would like it?***  1 = Yes, always  2 = Yes, for the most part  3 = No, I have no breaks  4 = No, I have no breaks with colleagues | |
|  | **arblamna**  VIP: 1991->  MONICA: 1990-2009 | ***Is your work of nature that you can leave it for a while if you want to speak with a colleague?***  1 = Yes, for the most part  2 = Yes, sometimes  3 = Only for urgent matters  4 = No, it is impossible | |
|  | **arbkontakt**  VIP: 1991->  MONICA: 1990-2009 | ***Have you, as a part of your work, a lot of contacts with your colleagues?***  1 = Yes, constantly work contacts  2 = A or a few times / month  3 = No, I work mostly alone  4 = Rarely or never | |
|  | **arbfritid**  VIP: 1991->  MONICA: 1990-2009 | ***How often do you usually together with one or more of your colleagues at the time?***  1 = One or more times / week  2 = A or a few times / month  3 = One or a few times / year  4 = Rarely or never | |
|  | **arbbesok**  VIP: 1991->  MONICA: 1990-2009 | ***When visiting any of your colleagues you later?***  1 = for one to four weeks  2 = for one to twelve months ago  3 = For more than a year ago  4 = Has never been visited by any colleague | |
|  | **MONICA_motion_fritid_86_09**  MONICA: 1986-2009 Applies only to participants of MONICA 1986-2009 | **How much have you moved you or exerted yourself physically in your leisure time in the past year?** 1 = hardly any at all  2 = mostly sedentary, sometimes someone walking or the like  3 = Light physical effort at least 2 hours a week.  4 = More strenuous exercise 1-2 hours a week.  5 = More strenuous exercise at least 3 hours a week.  6 = Hard training or racing regularly and several times a week where the physical effort is large.  7 = Never (only 1986)  8 = 1-2 times per month (only 1986)  9 = 1 time per week (only 1986)  10 = 2-3 times per week (only 1986)  11 = 4 or more times per week (only 1986) | |
|  | **MONICA_motion_arbete**  MONICA: 1990-2009 Applies only to participants of MONICA 1990-2009. No similar issue there in 1986. | **How much have you moved you or exerted yourself physically in your work in the past year?** 1 = Do old-age retirement (this option is not 1990)  2 = sedentary work  3 = Easy, but mobile work  4 = moderately heavy work  5 = Heavy work | |
|  | **g1_a-g1_d**  VIP: 1989->  MONICA: 2014  Applies only to participants in the VIP + MONICA 2014 | **Mark in the table below how you often travel to and from work each season** Spring, g1_a 1 = Car  2 = Bus  3 = Walking  4 = Bicycles  Summer, g1_b  1 = Car  2 = Bus  3 = Walking  4 = Bicycles  Autumn, g1_c  1 = Car  2 = Bus  3 = Walking  4 = Bicycles  Winter g1_d  1 = Car  2 = Bus  3 = Walking  4 = Bicycles  Double digits or greater indicates what options combined (only MONICA) | |
|  | **antal_km**  VIP: 1989->  MONICA: 2014  Applies only to participants in the VIP + MONICA 2014 | **Number of km to work (one way)** | |
|  | **g2_a - g2_e**  VIP: 1989->  MONICA: 2014  Applies only to participants in the VIP + MONICA 2014 | **Select the option that best describes your work** g2_a = sedentary or portrait  g2_b = Slight but partially movable  g2_c = Slight and movable  g2_d = Sometimes physically demanding  g2_e = physically demanding most of the time | |
|  | **g3_a**  VIP: 1989->  Applies only to participants in the VIP | ***How often are you in your spare time walking?***  0 = Never  1 = 1-2 / month  2 = 3-4 times / month  3 = 2-3 times / week  4 = Every day | |
|  | **g3_b**  VIP: 1989->  Applies only to participants in the VIP | ***How often are you in your spare time bike rides?***  0 = Never  1 = 1-2 / month  2 = 3-4 times / month  3 = 2-3 times / week  4 = Every day | |
|  | **g3_c**  VIP: 1989-2005  Applies only to participants in the VIP | ***How often are you in your free time to dance / folk dance?***  0 = Never  1 = Rare  2 = Occasionally each month  3 = Weekly  4 = Every day | |
|  | **g3_d**  VIP: 1989-2005  Applies only to participants in the VIP | ***How often are you in your spare time shoveling snow?***  0 = Never  1 = Rare  2 = Occasionally each month  3 = Weekly  4 = Every day | |
|  | **g3_e**  VIP: 1989-2005  Applies only to participants in the VIP | ***How often are you in your leisure time to gardening?***  0 = Never  1 = Rare  2 = Occasionally each month  3 = Weekly  4 = Every day | |
|  | **g3_f**  VIP: 1989-2005  Applies only to participants in the VIP | ***How often are you in your spare time hunting / fishing?***  0 = Never  1 = Rare  2 = Occasionally each month  3 = Weekly  4 = Every day | |
|  | **g3_g**  VIP: 1989-2005  Applies only to participants in the VIP | ***How often are you in your spare time to carry / mushroom picking?***  0 = Never  1 = Rare  2 = Occasionally each month  3 = Weekly  4 = Every day | |
|  | **g4**  VIP: 1989-2005  Applies only to participants in the VIP | **Have you changed your "daily exercise" in the past year?** 1 = very Reduced  2 = Reduced any  3 = As previously  4 = Increased slightly  5 = very Increased | |
|  | **G5**  VIP: 1989-2005  Applies only to participants in the VIP | **"The daily exercise I get to satisfy my need to touch me." Does this statement to you?** 1 = not at all  2 = rather poor  3 = Partial  4 = Completely | |
|  | **g6**  VIP: 1989->  MONICA: 2014  Applies only to participants in the VIP + MONICA 2014 | **How often have you been training or exercising in gym clothes the last three months, in order to improve your fitness and / or to feel good?** 1 = Never  2 = Occasionally - not regularly  3 = 1-2 times / week  4 = 2-3 times / week  5 = more 3 times / week | |
|  | **g7**  VIP: 1989-2005  Applies only to participants in the VIP | **If you exercise - you have changed your exercise habits over the past year?** 1 = very Reduced  2 = Reduced any  3 = As previously  4 = Increased slightly  5 = very Increased | |
|  | **g8**  VIP: 1989-2005  Applies only to participants in the VIP | **How physically active you were before 20 years of age?** 1 = Freed from school gymnastics  2 = Played only in school gymnastics  3 = Trained without competing  4 = Played the competition and training (not elite)  5 = trained and raced elite  Double digits or greater indicates what options combined. | |
|  | **G9**  VIP: 2005->  MONICA: 2014 | ***How much have you exerted yourself physically in the last 12 months?***  If your activity varies between eg summer and winter, so try to take an average.  1 = sedentary leisure.  You spend most of your time reading, television, cinema or other sedentary leisure. You walk, cycle on the move in other ways less than two hours a week.  2 = Moderate exercise at leisure.  You walk, cycle or move about in any other way for at least two hours a week without breaking a sweat. This included such as walking or cycling to and from work, other walks, heavy housework, normal gardening, fishing, table tennis, bowling.  3 = Moderate, regular exercise at leisure.  You exercise regularly 1-2 times per week for at least 30 minutes at a time with such running, swimming, tennis, badminton or other activity that makes you sweat.  4 = Regular exercise and training.  You participate eg running, swimming, tennis, badminton, gymnastics or the like at an average of at least three times per week. Each time lasts at least 30 minutes at a time. | |
|  | **g10**  VIP: 2005->  Applies only to participants in the VIP | ***How much time do you spend in a normal week do moderately strenuous activities that make you hot?***such as brisk walks, gardening, heavy housework, cycling, swimming. It can vary throughout the year, but try taking some kind of average.  1 = 5 hours a week or more  2 = more than 3 hours but less than 5 hours a week.  3 = between 1 to 3 hours per week.  4 = more than 1 hour a week.  5 = Not at all.  6 = Do not know / can not take a position. | |
|  | **g11a_h - g11b_ej**  VIP: 2011->  Applies only to participants in the VIP  These are questions of sedentary taken from the IPAQ (validated questions about physical activity). | ***How long have you per day during the last 7 days spent sitting in connection with work, studies and transport, at home and in your spare time?***  Try to estimate how many hours on average. Examples of this time at a desk, visiting friends, going by car or bus, to sit and eat or talk to sit at the computer and watching movies or TV.  Hours on weekdays, g11a_h  Number of minutes weekdays, g11a_m  Do not know weekdays, g11a_ej  Hours weekends, g11b_h  Minutes weekends, g11b_m  Do not know weekends, g11b_ej | |
|  | **exercise**  VIP: 1988-1991  For older polls VIP | **How much do you exercise in your spare time?** 0 = Virtually none  1 = Occasionally  2 = regularly about once a week  3 = Regular about two times a week  4 = Regular quite sharply at least twice a week | |
|  | **motion2**  VIP: 1986-1994  For older polls VIP | **How often do you exercise?** 1 = Never  2 = 1-2 / month  3 = 1 day / week  4 = 2-3 times / week  5 = 4 or more times / week | |
|  | **sleep_h7a - sleep_h7h**  VIP: 2005->  Applies only to participants in the VIP | ***How much risk is there that you would doze off or fall asleep in the following situations, as opposed to just feeling tired?***  This refers to your usual way of life in recent times. Even if you have not done all this recently, so try to think of how it would have affected you.  **sleep_h7a**, Sitting and reading.  1 = No  2 = Small  3 = moderate  4 = Large  **sleep_h7b**, Watching TV. (Same options as above)  **sleep_h7c**, Sitting, inactive in a public place (eg theater or a meeting). (Same options as above)  **sleep_h7d**, As a passenger in a car for an hour without break. (Same options as above)  **sleep_h7e**, Lying down and resting in the afternoon if conditions permit. (Same options as above)  **sleep_h7f**, Sitting and talking to someone. (Same options as above)  **sleep_h7g**, Sitting quietly after eating lunch (without alcohol). (Same options as above)  **sleep_h7h**In a car stopped a few minutes in traffic. (Same options as above) | |
|  | **sleep_h8a**  VIP: 2005->  Applies only to participants in the VIP | ***Do you snore when you sleep?***  1 = Yes, always  2 = Yes, almost always  3 = Yes, sometimes  4 = No almost never  5 = No never  6 = DK | |
|  | **sleep_h8b**  VIP: 2005->  Applies only to participants in the VIP | ***Does your wife / husband / partner noticed that you have pauses in breathing during sleep?***  1 = Yes, always  2 = Yes, almost always  3 = Yes, sometimes  4 = No almost never  5 = No never  6 = DK | |
|  | **CAGE**  Applies only to participants in the VIP | **CAGE (Cut down, annoyance, guilt Eye opener)**calculated based on the questions i2, i3, i4 and i5 alternatively J12, J11, J6 and J7. CAGE recalculated together into an index from 0-4 0-1 where interpreted as "not character to hazardous alcohol" and 2-4 interpreted as "possible hazardous alcohol / alcohol"  Note that the individuals who responded that they are absolutist on the issue i1 invited skip question i2-i5, meaning they lack the answers to these questions are not real missing. | |
|  | **AUDIT**  Applies only to participants in the VIP | **AUDIT (Alcohol Use Disorders Identification Test)**provides an indication of risky / harmful alcohol consumption or abuse / dependence. The index is calculated based on the questions J1-J10.  All questions in the AUDIT has a score ranges from 0-4 points. The answers to these questions have thus been scored and summed. The figures stand next to the answer options in the questionnaire is not the same as the points that answer gives. Maximum points is 40th  *For women:*  6 points or more indicates risky / harmful alcohol  14 points or more means likely abuse / dependence  *For men:*  8 points or more indicates risky / harmful alcohol  16 points or more means likely abuse / dependence | |
|  | **i1**  VIP: 1988-2005  Applies only to participants in the VIP | ***Are you a teetotaler?*** (From 1992, it has been asked to reply to question i2-i5 if you answered yes to i1. When we do not exclude existing answer may still be value to them.)  1 = Yes  2 = No | |
|  | **i2**  VIP: 1989->  Applies only to participants in the VIP | ***Have you ever thought you should reduce your alcohol consumption?***  1 = Yes  2 = No | |
|  | **i3**  VIP: 1989->  Applies only to participants in the VIP | ***Have people annoyed you by criticizing your drinking?***  1 = Yes  2 = No | |
|  | **i4**  VIP: 1989-2005  Applies only to participants in the VIP | ***Have you ever felt uncomfortable or felt guilty for the way you drink?***  1 = Yes  2 = No | |
|  | **i5**  VIP: 1989-2005  Applies only to participants in the VIP | ***Have you ever drunk first thing in the morning to calm nerves or cure a hangover (taken you a restorer)?***  1 = Yes  2 = No | |
|  | **J1**  VIP: 2005->  MONICA: 2014 | ***How often do you drink alcohol?***  1 = Never  2 = 1 time per month or less frequently  = 3 times in 2-4 months  4 = 2-3 times a week  5 = 4 times / week or more | |
|  | **j2**  VIP: 2005->  MONICA: 2014 | ***How many glasses do you drink on a typical day when you drink alcohol?***  With a glass mean:  50 cl beer  33 cl beer  1 glass of red or white wine  1 small glass fortified  4 cl spirits e.g. whiskey  1 = 0-2 glass  2 = 3-4 glass  3 = 5-6 glass  4 = 7-9 glass  5 = 10 or more glass | |
|  | **J3**  VIP: 2005->  MONICA: 2014 | ***How often do you have six glasses or more at the same time?***  1 = Never  2 = Less often than once a month  3 = Monthly  4 = Weekly  5 = daily or almost daily | |
|  | **J4**  VIP: 2005->  Applies only to participants in the VIP | ***How often during the last year have you been unable to stop drinking once you started?***  1 = Never  2 = Less often than once a month  3 = Monthly  4 = Weekly  5 = daily or almost daily | |
|  | **J5**  VIP: 2005->  Applies only to participants in the VIP | ***How often during the last year have you failed to do something which you should because you drank?***  1 = Never  2 = Less often than once a month  3 = Monthly  4 = Weekly  5 = daily or almost daily | |
|  | **J6**  VIP: 2005->  Applies only to participants in the VIP | ***How often during the last year have you needed a drink in the morning after drinking the day before (taking a restorer)?***  1 = Never  2 = Less often than once a month  3 = Monthly  4 = Weekly  5 = daily or almost daily | |
|  | **J7**  VIP: 2005->  Applies only to participants in the VIP | ***How often during the last year have you had feelings of guilt or remorse after drinking?***  1 = Never  2 = Less often than once a month  3 = Monthly  4 = Weekly  5 = daily or almost daily | |
|  | **J8**  VIP: 2005->  Applies only to participants in the VIP | ***How often during the last year have you had so you day after unable to remember what you said or did?***  1 = Never  2 = Less often than once a month  3 = Monthly  4 = Weekly  5 = daily or almost daily | |
|  | **J9**  VIP: 2005->  Applies only to participants in the VIP | ***Have you or someone else been injured because of your drinking?***  1 = No  2 = Yes, but not in the last year  3 = Yes, in the past year | |
|  | **J10**  VIP: 2005->  Applies only to participants in the VIP | ***Have a relative or friend, a doctor (or other healthcare) concerned about your drinking or suggested you cut down?***  1 = No  2 = Yes, but not in the last year  3 = Yes, in the past year | |
| Rökdata | *Certain assumptions have been made to correct and interpret the raw data, taking into account the survey design.* | | |
|  | **sm_status**  VIP: 1985->  MONICA: 1986-2014 | ***Smoking status:***  1 = Smokers  2 = Ex smoking  3 = Non Smoking  4 = smoke occasionally  5 = Smoked previously and then | |
|  | **sm_cig_groups**  VIP: 1992->  Applies only to participants in the VIP | ***Number of cigarettes per day:***  1 = 1-4  2 = 5-14  3 = 15-24  4 => 25 | |
|  | **sm_num_cig**  VIP: 1985-1992  MONICA: 1986-2014 | ***Number of cigarettes per day*** | |
|  | **sm_num_cigar**  VIP: 1985-1992  MONICA: 1986-2009 | ***Number of cigars per day***  *(Because of that, in some survey types requested cigars per week, the number in some cases divided by 7)* | |
|  | **sm_gr_tobacco**  VIP: 1985-1992  MONICA: 1986-2014 | ***Grams of tobacco per week*** | |
|  | **sm_how_often**  VIP: 2011->  MONICA: 1994-2014 | ***How often do you smoke?***  *(Is a supplementary to those who answered "Yes, I smoke occasionally not every day")*  1 = less than 1 day / month  2 = 1 to 3 days / month  Usually, 3 = 1 day / week  4 = Usually 2 to 4 days / week  5 = Almost every day  *Note: The MONICA, only options 3-5.* | |
|  | **sm_start**  VIP: 1985->  MONICA: 1986-2014 | ***Age at rökstart***  *Note: Not available 1986* | |
|  | **sm_stop**  VIP: 1985->  MONICA: 1986-2014 | ***Age at cessation of smoking*** | |
|  | **sm_duration**  VIP: 1985->  MONICA: 1986-2014 | ***Smoked in the number of years calculated from sm_start and sm_stop***  For those who indicated that they smoke and value for sm_stop lacking indicated sm_duration up until the date of the survey. Please note that the information in sm_duration can be misleading for the group smokers indicated that they have ever stopped smoking (and then started again). | |
|  | **sm_whystop_1**  VIP: 1988-1992  MONICA: 1990-2009 | ***Quit smoking for health reasons, on its own initiative***  1 = Yes | |
|  | **sm_whystop_2**  VIP: 1988-1992  MONICA: 1990-2009 | ***Quit smoking on the advice of a doctor / medical staff***  1 = Yes | |
|  | **sm_whystop_3**  VIP: 1988-1992  MONICA: 1990-2009 | ***Quit smoking because. other information / education***  1 = Yes | |
|  | **sm_whystop_4**  VIP: 1988-1992  MONICA: 1990-2009 | ***Quit smoking because. pressure from peers / family***  1 = Yes | |
|  | **sm_whystop_5**  VIP: 1988-1992  MONICA: 1990-2009 | ***Quit smoking for other reasons***  1 = Yes | |
|  | **sn_status**  VIP: 1985->  MONICA: 1986-2014 | ***Snuff Status:***  1 = snuff user  2 = Ex snus  3 = Non snus | |
|  | **sn_quantity**  VIP: 1985->  MONICA: 1986-2014 | ***Heavy user number snuff boxes per week***  1 = less than 2  2 = 2 to 4  3 = more than 4 but less than 7  4 = 7 or more | |
|  | **sn_time**  VIP: 1988->  MONICA: 1990-2014 | ***Used snus number of years*** | |
|  | **sn_stopsmoke_a**  VIP: 1991->  MONICA: 1990-2014 | ***Began snorted when you quit smoking:***  1 = Yes  2 = No  3 = Both smoking and snuff | |
|  | **sn_stopsmoke_b**  VIP: 1988-1992  Applies only to participants in the VIP | ***Began snorted when you quit smoking:***  1 = Yes  2 = No | |
|  | **sn_nicotine_replace**  VIP: 2006->  Applies only to participants in the VIP | **Did you use nicotine replacement therapy to become snusfri?** 1 = Yes  2 = No | |
|  | **sm_nicotine_replace**  VIP: 2006->  Applies only to participants in the VIP | **Did you use nicotine replacement therapy to quit smoking?** 1 = Yes  2 = No | |
|  | **nicotine_replace**  VIP: 2006->  Applies only to participants in the VIP | **Still using medicines to treat nicotine addiction, even though you do not smoke or use snuff anymore?** 1 = Yes  2 = No | |
|  | **nicotine_94_09**  MONICA: 1994-2009  Applies only to participants of MONICA | **Do you use daily second nicotine products?** 1 = Yes, chewing  2 = Yes, nicotine (nicotine gum patch is, -nässpray)  3 = Other  4 = No  *Note: Except for 1986 and 1990* | |
|  | **sm_yes_no**  VIP: 1985-2010 | **Smoking (for those who have rökdata VIP-poll)** 0 = Non-smokers / no response  1 = Smokers | |
|  | **sn_yes_no**  VIP: 1985-2010 | **Snus (for those who have rökdata VIP-poll)**0 = Non-snus / no response 1 = snuff user | |
